# Reconstitution of Membrane Protein Complexes Involved in Pneumococcal Septal Cell Wall Assembly

**DOI:** 10.1371/journal.pone.0075522

**Published:** 2013-09-23

**Authors:** Marjolaine Noirclerc-Savoye, Violaine Lantez, Luca Signor, Jules Philippe, Thierry Vernet, André Zapun

**Affiliations:** 1 Institut de Biologie Structurale, Université Grenoble Alpes, Grenoble, France; 2 Institut de Biologie Structurale, Direction des Sciences du Vivant, Commissariat à l’energie atomique et aux Energies Alternatives, Grenoble, France; 3 Institut de Biologie Structurale, Centre National de la Recherche Scientifique, Grenoble, France; Centre National de la Recherche Scientifique, Aix-Marseille Université, France

## Abstract

The synthesis of peptidoglycan, the major component of the bacterial cell wall, is essential to cell survival, yet its mechanism remains poorly understood. In the present work, we have isolated several membrane protein complexes consisting of the late division proteins of *Streptococcus pneumoniae*: DivIB, DivIC, FtsL, PBP2x and FtsW, or subsets thereof. We have co-expressed membrane proteins from *S. pneumoniae* in *Escherichia coli*. By combining two successive affinity chromatography steps, we obtained membrane protein complexes with a very good purity. These complexes are functional, as indicated by the retained activity of PBP2x to bind a fluorescent derivative of penicillin and to hydrolyze the substrate analogue S2d. Moreover, we have evidenced the stabilizing role of protein-protein interactions within each complex. This work paves the way for a complete reconstitution of peptidoglycan synthesis *in vitro*, which will be critical to the elucidation of its intricate regulation mechanisms.

## Introduction

Bacterial cell division requires robust mechanisms to maintain the cell shape. Although numerous proteins participating in cell division and wall assembly have been identified and localized in model bacteria, the fundamental processes at play are still poorly understood and *in vitro* reconstitutions are experimentally challenging [1-3]. The shape of bacteria is determined by that of their cell wall and its major component, the peptidoglycan (PG), which is made of glycan chains cross-linked by short stem peptides [4-6]. The glycosyltransferase (GT) domain of penicillin-binding proteins (PBPs) catalyses the polymerization of the glycan strands (transglycosylation), whereas the transpeptidase (TP) domain is responsible of the cross-linking of the stem peptides (transpeptidation) [2,6-10]. *In vitro* complete PG assembly resulting from both GT and TP activities has never been achieved with Gram-positive enzymes, but only with recombinant PBPs from *Escherichia coli* [11-13]. This is possibly due to the improper nature of the peptide stem available [2,14], or to missing interacting partners.

The human pathogen *Streptococcus pneumoniae* contains three GT/TP bifunctional class A PBPs (PBP1a, PBP1b and PBP2a) and two TP monofunctional class B PBPs (PBP2b and PBP2x) [15-17]. In ovococcus bacteria such as streptococci, two types of PG assembly have been proposed to contribute to cell wall formation [18,19]. One machinery would carry out peripheral PG synthesis, whereas the other would synthesize septal PG, both including one specific TP monofunctional class B PBP. The participation of class A PBPs to either type of PG assembly is unknown, while it has been recently proposed that PBP1a could be present in both machineries [20,21]. Additionally, conserved division or morphogenetic proteins [15,16] as well as various peptidoglycan hydrolases are believed to participate to septal and peripheral machineries (reviewed in 3,22). However, the fact that the two machineries form two separate complexes or are assembled into a single large one is not clear, although the existence of one large assembly is now favored [19,22].

The complex responsible of septal PG synthesis would comprise DivIB, FtsL, DivIC, FtsW in addition to the TP PBP2x [18,23]. The role of DivIB, FtsL, DivIC in the synthesis or remodelling of septal PG is inferred from their essentiality for cell division in most organisms, and their absence from bacteria without cell walls [24]. These three bitopic membrane proteins appear to bridge cytoplasmic and periplasmic cell division proteins [25,26]. The 10-trans-membrane (TM) segment protein FtsW [27] translocates the lipid-linked PG precursor across the cytoplasmic membrane [28]. Interactions between DivIB, FtsL, DivIC, FtsW and PBP2x have been largely suggested from *in vivo* experiments performed in *E. coli* and *Bacillus subtilis* [29-33]. However, the failure to purify such complexes *in vivo* [29] suggests that they are either labile or transient and regulated [31,34].

*In vitro* reconstitution of septal PG synthesis pertains both to cell wall assembly and bacterial cell division. In this study, we have focused on the full length membrane proteins recruited to midcell, including DivIB, DivIC, FtsL, FtsW and PBP2x, as their TM domains are essential to their recruitment at the division site and likely involved in protein-protein interactions [35-40]. Although the membrane character of proteins greatly increases the difficulty to achieve reconstitution, we successfully purified four membrane protein complexes containing from two to five different membrane proteins. The characterization of these complexes allowed identification of protein-protein interactions consistent with published *in vivo* experiments and the TP reporter activity of PBP2x validated the functionality of the purified complexes. The reconstitution of protein complexes involved in the PG synthesis process constitutes a first step towards an understanding of cell division and cell wall formation events in eubacteria.

## Materials and Methods

### Plasmids construction

The plasmids used for over-expression of membrane proteins are described in Table 1. Molecular cloning, PCRs, and *E. coli* transformations were carried out as previously described [41].

**Table 1:** Plasmids used in this study.

**Plasmids**	**Overexpressed membrane protein(s**)** from MCS1**	**Overexpressed membrane protein(s**)** from MCS2**
pMN2 (AmpR)^a^	DivIC, FtsL, H-DivIB	None
pMN14 (AmpR)^a^	DivIC, FtsL, DivIB	H-FtsW, PBP2x-S
pMN15 (AmpR)^a^	DivIC, FtsL, DivIB	FtsW, PBP2x-S
pMN18 (AmpR)^a^	DivIC, FtsL, H-DivIB	PBP2x-S
pMN19 (AmpR)^a^	DivIC, FtsL, H-DivIB	PBP2b-S
pMN20 (AmpR)^a^	DivIC, FtsL, H-DivIB	FtsW-S
pMN22 (AmpR)^a^	H-DivIB	PBP2x-S
pETDuet-HisPatAB (AmpR)^a^	H-PatA	H-PatB
pET30-HDivIB (KanR)^b^	H-DivIB	NA
pET30-HPBP2x (KanR)^b^	H-PBP2x	NA
pET30-PBP2xS (KanR)^b^	PBP2x-S	NA
pET30-HFtsW (KanR)^b^	H-FtsW	NA
pET30-GSTDivIB* (KanR)^b^	GST-DivIB*	NA
pET30-GSTPBP2x* (KanR)^b^	GST-PBP2x*	NA

^a^ derivative of pETDuet (Novagen); ^b^ derivative of pET30b (Novagen), * extracellular domain

Genes encoding full length membrane proteins were amplified from *S. pneumoniae* R6 chromosomal DNA and introduced in modified pET-30 plasmids or in the multiple cloning sites MCS1 and MCS2 of the pETDuet vector (Novagen). For single His-tagged membrane proteins, genes were first introduced as *Nco*I-*Bam*HI fragments into pETDuet-H8N (lab collection), derivative of pETDuet. For single Strep-tagged membrane proteins, amplified genes were first introduced individually as *Nde*I-*Bam*HI fragments into pET30-StpN (lab collection), a derivative of pET30 (Novagen).

Bi- or tricistonic operons were constructed by taking advantage of a feature of these vectors, which contain a *Xba*I site between the promoter and the ribosome binding site (RBS) upstream of the MCS (MCS1 in pETDuet). Providing that a first gene was introduced with a *Spe*I or *Nhe*I additional restriction after its stop-codon, a second gene excised from a pET30 or pETDuet MCS1, using *Xba*I and another site downstream of the ORF, can be introduced with its RBS downstream of the first gene. This is possible since *Xba*I produces overhangs compatible with the *Nhe*I and *Spe*I sites. The ligation of the *Xba*I site, with the *Nhe*I or *Spe*I sites is destructive, so that the procedure can be repeated to add further genes to the operon.

### Expression and purification of recombinant membrane protein complexes

Membrane proteins were overexpressed in *E. coli* BL21 (DE3) cells, in Luria Bertani broth. Expression was induced with 1 mM IPTG (at an optical density at 600 nm of 2 to 2.5) and performed overnight at 20°C. Bacteria were collected by centrifugation (3000*g*, 15 min, 4°C) and resuspended in 50 mM HEPES pH 7.5, 50 mM NaCl, 10% glycerol (HNG buffer) with protease inhibitors (Complete, Roche). Cell lysis was carried out using a Microfluidizer™ (Microfluidics) at 10 000 psi. After clarification by centrifugation (40 000*g*, 20 min, 4°C), membranes were collected by ultracentrifugation (180 000*g*, 1 h, 4°C) and resuspended in the HNG buffer. Membranes were solubilized with 10 mM *n*-dodecyl-β-D-maltopyranoside (DDM) (1 hour, 4°C) and solubilized proteins were recovered in the supernatant after ultracentrifugation (180 000*g*, 30 min, 4°C).

The solubilized membrane proteins and complexes were isolated by one or two successive affinity chromatographies. Solubilized proteins were incubated overnight with Ni-NTA matrix (Qiagen). After washing steps with HNG buffer containing 50 mM imidazole and 10 mM DDM (2 column volumes (CV)), 2 mM DDM (2 CV) and 0.3 mM DDM (4 CV), respectively, bound proteins were eluted with 300 mM imidazole. Fractions containing Strep-tagged co-eluted proteins were loaded onto Strep-Tactin matrix (IBA lifesciences). After washing with HNG buffer containing 0.3 mM DDM (5 CV), purified proteins were eluted with 2.5 mM desthiobiotin. Protein samples were analyzed by SDS-PAGE using 14.5% acrylamide gels (acrylamide/bisacrylamide ratio: 37.5/1) and a Tris/Tricine buffer system, at 150 V with a cooling system (15-18 °C). DDM-purified membrane complexes were concentrated by ultrafiltration (Amicon 100 kDa cutoff, Millipore).

The extracellular domains of PBP2x (PBP2x*) and DivIB (DivIB*) fused to the GST tag or H-PatAB were overexpressed and purified as previously described [23,26,42].

### Liquid Chromatography Electrospray Ionization Mass Spectrometry (LC/ESI-MS)

LC/ESI-MS was performed on a 6210 LC-TOF spectrometer coupled to a HPLC system (Agilent Technologies). Samples were desalted on a protein macroTrap (reverse phase-C4, Michrom Bioresources) for 3 min at 100 µl/min with 100% solvent A (0.03% trifluoroacetic acid in H_2_0). The reverse phase-C4 column (Jupiter, 5 µm, 300 Å, 1 mm ID×50 mm, Phenomenex) was eluted at 50 µl/min with a linear gradient from 5% to 95% of solvent B (95% acetonitrile, 5% H_2_0, 0.03% trifluoroacetic acid) in 15 min. MS acquisition was carried out in the positive ion mode in the 300-3000 *m/z* range and the data processed with MassHunter software (v. B.02.00, Agilent Technologies).

### In-gel digestion and peptide mass fingerprinting by MALDI TOF mass spectrometry

Selected bands were in-gel digested as previously described [43] in presence of 0.01% ProteaMAX surfactant (Promega). Mass spectra of the tryptic peptides were recorded on an Autoflex mass spectrometer (Bruker, Bremen, Germany) in the reflectron positive ion mode detection. Samples were desalted and concentrated on RP-C18 tips (Millipore) and eluted directly with 2 µl of α-cyano-4-hydroxy cinnamic acid matix (10 mg/ml in H_2_0/acetonitrile/trifluoroacetic acid: 50/50/0.1) on the target.

### Characterization of the purified membrane protein complexes

Size exclusion chromatography analyses were performed on Superdex 200 10/30 or Superose 6 16/60 columns (GE Healthcare) in HNG buffer containing 0.3 mM DDM.

Trypsin digestions were performed at 25°C, with an estimated proteomic grade trypsin (Sigma) to membrane protein ratio of 1:100. The concentration of purified H-DivIB alone was determined by its absorbance at 280 nm. The amount of each complex used for the trypsin digestion was then determined by densitometry following Coomassie-stained SDS-PAGE in order to include about 0.5 µg of H-DivIB. Aliquots were collected after 15, 30, 60 and 120 min of trypsin digestion and analysed by Coomassie-stained SDS-PAGE.

Quantification of PBP2x active site and determination of kinetic parameter

Quantification of PBP2x active site was performed by adding 10 µmole of Bocillin FL (Molecular probe) to 4 to 7 10^-12^ mole of PBP2x. The labeled proteins were visualized after SDS-PAGE with a Geldoc XR fluorescent imager (Biorad) using a 530 nm excitation light. The fluorescence intensities were quantified with Image Lab Software (Biorad). Standard curve was established with soluble-PBP2x (PBP2x*), purified as previously described [23]. The activity of PBP2x was assayed by measuring its ability to hydrolyze the S2d thioester analog of cell wall stem peptides as previously described [44,45]. The assay was performed in a 96-well plate format at 37°C in the presence of 50 mM potassium phosphate (pH 7.0), 2.0 mM of S2d, 3.2 mM dithiodipyridine, and 4 to 7 10^-12^ mole of PBP2x (0.7 to 1.2 10^-7^ M). The increase of absorbance at 330 nm was monitored with a FLUOstar Optima microplate reader (BMG labtech).

## Results and Discussion

### Purification of membrane protein complexes

Expressing all the membrane proteins of the septal PG machinery from *S. pneumoniae* in a single strain of *E. coli* would likely produce a mixture of complexes and sub-complexes that could be difficult to separate and isolate. To identify protein-protein interaction and isolate non-labile membrane protein complexes involving DivIB, DivIC, FtsL, PBP2x or FtsW, we fused His- and Strep-tags to two of the proteins (denoted with H- prefixes or -S suffixes). We then co-expressed various combinations of the 5 membrane proteins in single *E. coli* strain and solubilized the membranes with detergent. Stable recombinant complexes formed spontaneously in the cells were then purified by two successive chromatographic steps.

Several detergents were first screened to identify those preserving the integrity of the complexes (Figure S1). Although the H-DivIB/DivIC/FtsL complex was successfully purified in several detergents, DDM was selected for larger scale experiments due to its compatibility with mass spectrometry measurements [46].

**Figure 1:**
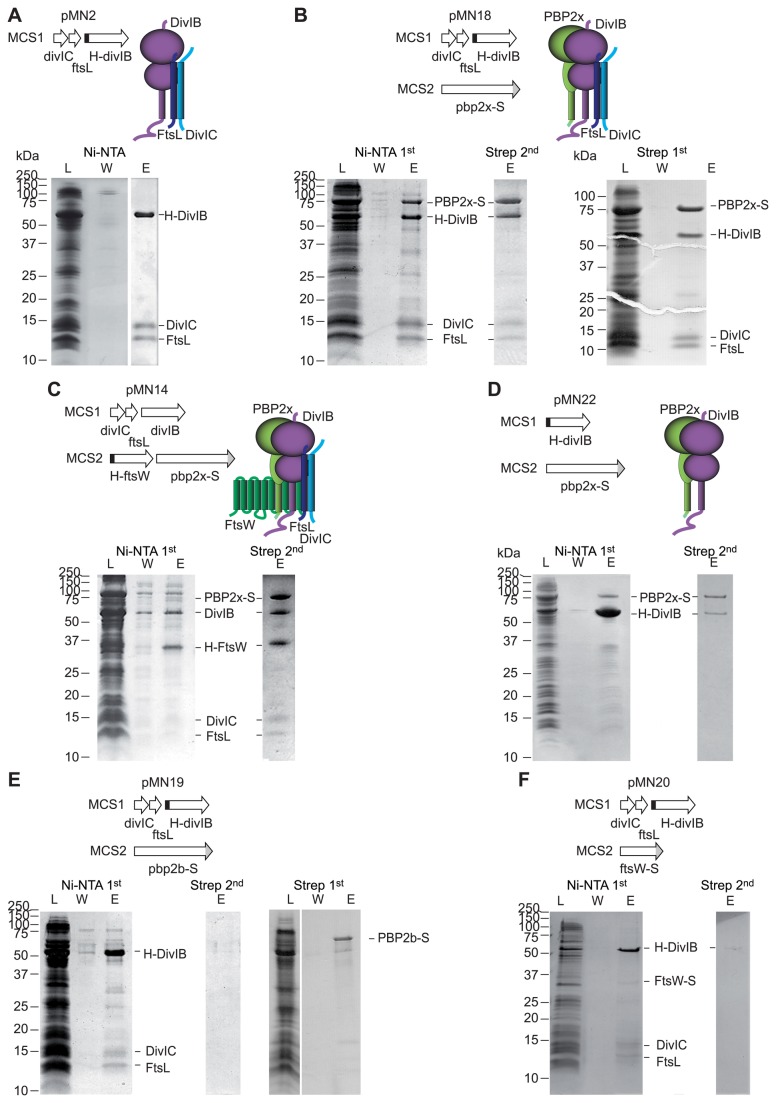
Purification of recombinant membrane protein complexes involved in septal cell wall synthesis. Membrane preparations of *E. coli* strains overexpressing different combinations of pneumococcal membrane proteins were subjected to detergent solubilization, Ni-NTA and Strep-Tactin affinity chromatography, as first or second steps. L, W and E stand for load, wash and elution fractions, respectively. Samples were analysed by Coomassie-stained SDS-PAGE. Over-expressed proteins were: **A**. H-DivIB, DivIC and FtsL; **B**. H-DivIB, DivIC, FtsL and PBP2x-S; **C**. DivIB, DivIC, FtsL, PBP2x-S and H-FtsW; **D**. H-DivIB, and PBP2x-S; **E**. H-DivIB, DivIC, FtsL and PBP2b-S; **F**. DivIB, DivIC, FtsL, PBP2x-S and FtsW.

Ni-NTA metal-affinity chromatography was performed with every solubilized extract and samples were analyzed by SDS-PAGE (Figure 1). The co-elution of proteins without His-tag from the Ni-NTA matrix, together with the absence of detected protein in the last washing step, was interpreted as evidence of complexes. These protein interactions were further confirmed by co-purifying the same proteins in a second Strep-tag affinity chromatography (Figure 1). Among all the combinations of over-expressed protein tested, we successfully isolated four membrane protein complexes: H-DivIB/DivIC/FtsL (Figure 1A); PBP2x-S/H-DivIB/DivIC/FtsL (Figure 1B); PBP2x-S/H-FtsW/DivIB/DivIC/FtsL (Figure 1C); and PBP2x-S/H-DivIB (Figure 1D). Note that H-DivIB/DivIC/FtsL and PBP2x-S/H-DivIB/DivIC/FtsL were also purified in a similar manner in Triton X-100 instead of DDM.

Purification performed from membranes co-expressing H-DivIB with DivIC and FtsL, revealed that both DivIC and FtsL were co-eluted with H-DivIB, while neither DivIC nor FtsL were tagged (Figure 1A). Note that for this complex, the second Strep-tag affinity purification step was omitted due to the absence of a Strep-tag on any protein. Due to the interaction of C-terminal extremities of DivIC and FtsL with the β-domain of DivIB [47], we did not attempt to fuse tags at the C-terminus of DivIC or FtsL. When a Strep-tag was fused to the N-terminus of DivIC, it was cleaved in the cells and did not allow affinity chromatography.

Ni-NTA purification from solubilized membranes of cells co-expressing PBP2x-S with H-DivIB, DivIC and FtsL, allowed co-elution of PBP2x-S, DivIC and FtsL with H-DivIB, whereas H-DivIB, DivIC and FtsL were co-purifed with PBP2x-S on Strep-Tactin, either as first or second chromatography step (Figure 1B).

From cells overexpressing H-FtsW, PBP2x-S, with un-tagged DivIB, DivIC and FtsL, we detected the five proteins both in Ni-NTA and Strep-Tactin elution fractions (Figure 1C). To our knowledge, no other complex of recombinant membrane proteins of such complexity had been isolated previously. This co-purification demonstrates that bacterial membrane protein complexes comprising large numbers of subunits can be produced recombinantly for biochemical studies.

Purification performed from cells overexpressing H-DivIB and PBP2x-S, in the absence of DivIC and FtsL, allowed the co-purification of PBP2x-S with H-DivIB. The large excess of H-DivIB over PBP2x-S in elution fractions from Ni-NTA chromatography was eliminated by the subsequent Strep-Tactin purification (Figure 1D, Figure S2). The role of the TM domains of both proteins in their mutual interaction was tested by co-purification experiments of the extracellular domain of PBP2x fused to the glutathione-S-transferase (GST-PBP2x*) with full length H-DivIB, or reciprocally of the extracellular domain of DivIB (GST-DivIB*) and full length PBP2x-S. Ni-NTA chromatography of a sample containing H-DivIB and GST-PBP2x* did not co-purify GST-PBP2x* significantly. A small amount of GST-PBP2x* was detected in the elution fraction, but it was similarly detected in the absence of H-DivIB, indicating a non-specific interaction of GST-PBP2x* with the Ni-NTA matrix (Figure S3A and S3B). No H-DivIB was retained by a subsequent glutathione affinity chromatography. Conversely, Strep-Tactin chromatography of solubilized membranes containing PBP2x-S and GST-DivIB* purified only PBP2x-S (Figure S3C). These results show that the TM segments of PBP2x and DivIB contribute to their interaction.

The identity of each protein in the complexes was verified by Western blots using anti-His-tag, anti-Strep-tag or specific antibodies (Figure S4). Masses predicted and those determined by electrospray mass spectrometry performed on purified complexes are in agreement, with the exception of that of multitopic FtsW, which could not be measured precisely. The mass of full length PBP2x-S (84 742.6 Da) was measured to be 84 742.81 ± 3.45 Da. All other proteins had their N-terminal methionine cleaved. The masses of H-DivIB (46 422.8 Da) and untagged DivIB (45 268.6 Da) involved in the pentameric complex, were measured to be 46 422.46 ± 1.45 Da and 45 270.79 ± 1.06 Da, respectively. The masses observed for DivIC (14 730.9 Da) and FtsL (12 074.9 Da) were 14 730.96 ± 1.09 Da and 12 075.59 ± 0.41 Da, respectively.

The specificity of the protein interactions revealed by co-purification was demonstrated by the absence of protein in elution fractions from a Ni-NTA chromatography when none of the five overexpressed membrane proteins carried a His-tag (Figure S5A). Also, when the other class B PBP from *S. pneumoniae* PBP2b was overexpressed with a C-terminal Strep-tag, it was not co-purified with H-DivIB/DivIC/FtsL, unlike PBP2x-S. We could only detect a very low amount of PBP2b-S in elution fractions of the Ni-NTA purification (Figure 1E) compared to PBP2x-S (Figure 1B). Further Strep-Tactin purification of the Ni-NTA elution fraction did not yield any detectable complex. Alternatively, when Strep-Tactin purification was carried out as first step, almost no H-DivIB/DivIC/FtsL was copurified with PBP2b-S, in contrast to what was observed with PBP2x-S (compare panels E and B in Figure 1). PBP2b is the class B PBP proposed to be specific of the peripheral PG synthesis machinery [18,48]. The different behaviour of PBP2x-S and PBP2b-S with respect to H-DivIB/FtsL/DivIC is consistent with the hypothesis of two specific complexes responsible of cell wall synthesis in *S. pneumoniae*.

When overexpressing FtsW-S, together with H-DivIB, DivIC and FtsL without a tag, we detected only small amounts of FtsW-S in elution fractions containing H-DivIB, DivIC and FtsL after Ni-NTA chromatography (Figure 1F). The subsequent Strep-Tactin purification of the Ni-NTA elution fraction did not yield any detectable complex. This result suggests that FtsW does not interact with DivIB, DivIC and FtsL proteins in the absence of PBP2x. However, as FtsW is weakly over-expressed in the cell membranes we must be cautious with this interpretation.

Although the interaction between the extracellular domains of DivIB, DivIC and FtsL had been previously demonstrated [26], this is to our knowledge the first time that the ternary complex is reconstituted and purified using recombinant full length membranes proteins. This detergent-solubilized complex is less labile than the one composed of the soluble extracellular domains [26,47], suggesting that the TM segments interact and stabilize the whole structure. Alternatively, the TM segments could help proper folding of the proteins, which would then better interact. This is consistent with the importance of the TM segments in the localization of these proteins [22,35-40].

The co-purification of PBP2x-S with H-DivIB/DivIC/FtsL demonstrates the formation of a complex that was previously suggested by Robichon et al [29]. In the absence of DivIC and FtsL, H-DivIB and PBP2x-S can also form a complex consistent with the demonstration in *B. subtilis* that these two proteins interact directly through their extra-cytoplasmic domains [49]. However, the failure to co-purify DivIB and PBP2x when one of their TM segments is missing, emphasizes the role of the TM segments in their interaction, as hinted by genetic and localization studies [36,37,50]. Furthermore, the lower yield of co-purification of PBP2x-S with H-DivIB alone compared to with H-DivIB/DivIC/FtsL (Figure 1D and 1B, respectively and Figure S2) suggests that the PBP2x-S/H-DivIB complex is stabilized by interaction with the DivIC/FtsL dimer. These observations combined with the localization of the *pbp2x* gene downstream of *ftsL* in most genomes [51], suggest an interaction between PBP2x and FtsL.

The successful purification of the complex comprising DivIB, DivIC, FtsL, PBP2x-S and H-FtsW, whereas no FtsW-S was co-purified with the H-DivIB/DivIC/FtsL complex, indicates a direct contact between PBP2x-S and H-FtsW. Overall, the co-purification results are consistent with the model of topological interrelationships between the divisomal proteins DivIB, DivIC, FtsL, FtsW and PBP2x orthologous proteins of *B. subtilis* proposed by Wadsworth et al [35].

Purified complexes, as well as DivIB alone (Figure S5B), were analysed by size exclusion chromatography on Superdex 200 and Superose 6 matrixes in the presence of DDM (Figure S6). We observed that none of the samples contained large aggregates that were excluded from the matrixes. H-DivIB (46.5 kDa) showed an apparent molecular mass of 320 kDa. Complexes had apparent molecular masses ranging from 320 to 1 940 kDa, increasing from H-DivIB/DivIC/FtsL to that comprising the five proteins. The presence of all expected proteins in the elution peaks was checked by immuno-blotting. Interpreting these large apparent molecular masses is however challenging, as the extracellular domain of DivIB, DivIC, FtsL, PBP2x are elongated [23,47,52,53] and the estimation of the molecular mass of proteins from their hydrodynamic properties in solution is not suitable for detergent-solubilized membrane proteins [54]. However, DivIB and the H-DivIB/DivIC/FtsL complex eluted in two peaks, indicating that these entities could form dimers. Self-association of DivIB was reported previously in a two-hybrid system providing that the cytoplasmic and TM regions were present [33]. The dimerization of DivIB/DivIC/FtsL has also been proposed by modelling studies [55].

### Limited proteolysis of membrane protein complexes

**Figure 2:**
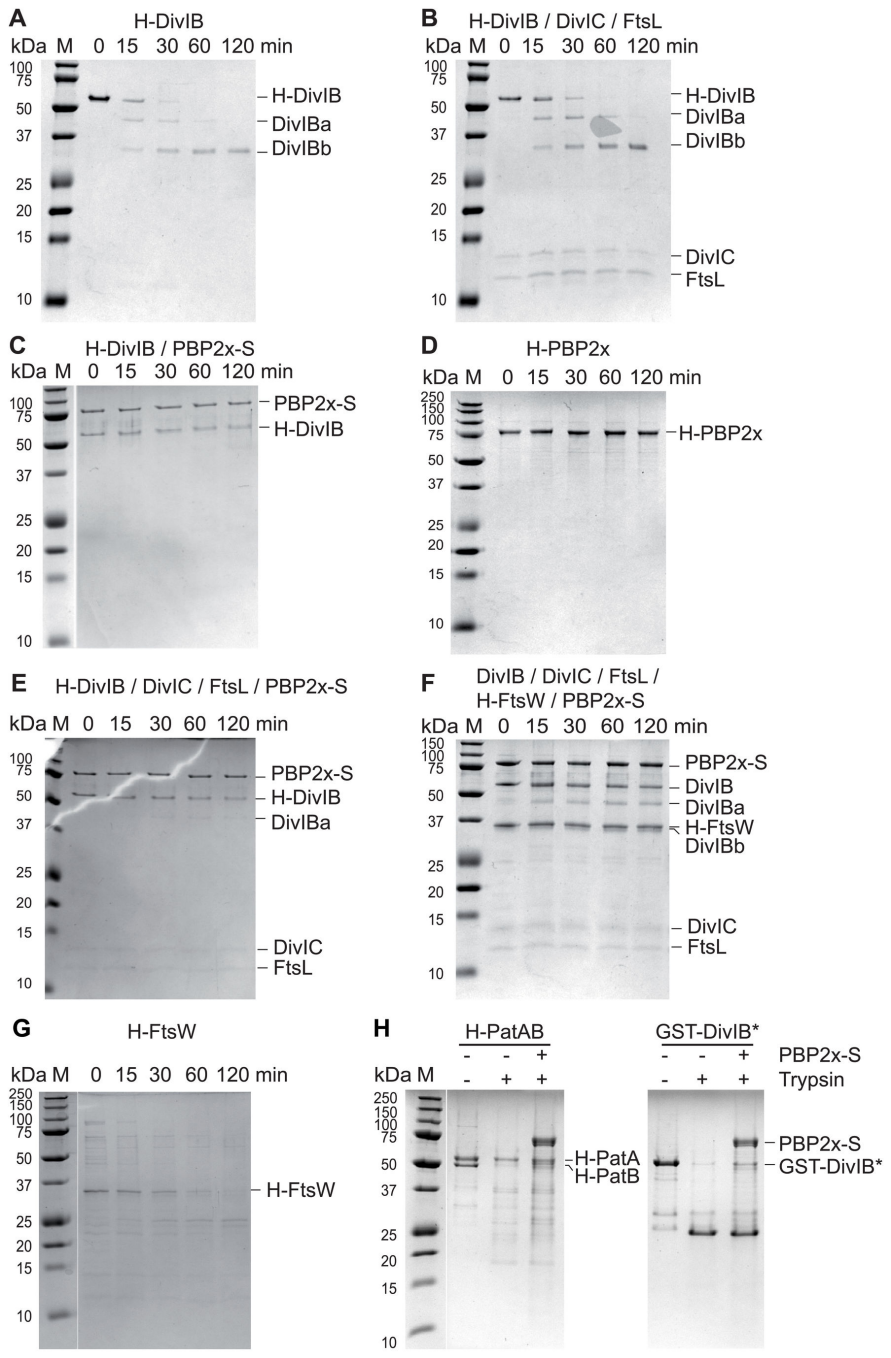
Comparative tryptic digestion of membrane protein complexes. Purified membrane proteins or complexes were incubated with trypsin at a 100/1 (w/w) ratio. Aliquots collected after 15, 30, 60 and 120 min were analysed by Coomassie-stained SDS-PAGE. **A**. H-DivIB; **B**. H-DivIB, DivIC and FtsL; **C**. H-DivIB, and PBP2x-S; **D**. H-PBP2x; **E**. H-DivIB, DivIC, FtsL and PBP2x-S; **F**. DivIB, DivIC, FtsL, PBP2x-S and H-FtsW; **G** H-FtsW; **H**. H-PatAB or GST-DivIB* +/- PBP2x-S after 30 min of incubation.

To gain clues on interacting regions, we compared the effect of trypsin digestion on H-DivIB, PBP2X-S and H-FtsW alone and engaged in the different complexes (Figure 2, Figure S5B-D). Time courses of digestion monitored by Coomassie-stained SDS-PAGE revealed a relative overall resistance to trypsin digestion of all three membrane proteins and cognate complexes, indicating that the recombinant proteins were folded in detergent.

**Figure 3:**
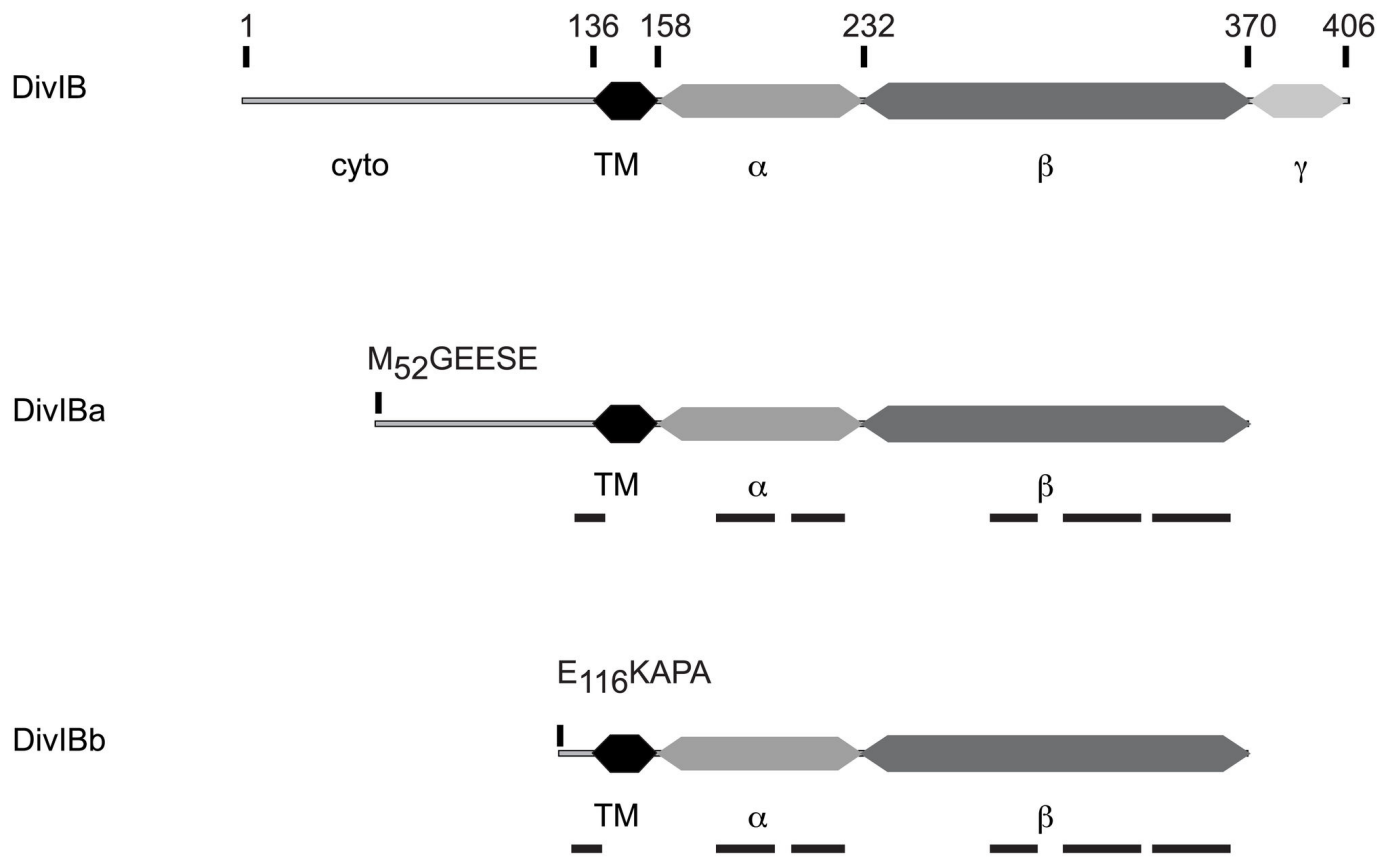
Schematic representation of the domain architecture of *S. pneumoniae* DivIB. Cyto and TM refer to the cytoplasmic domain and transmembrane segment. The three extracytoplasmic domains are designated α, β and γ. DivIBa and DivIBb are the intermediate and stable fragments obtained by trypsin digestion of DivIB. The bold lines represent the coverage of peptides founded by mass spectrometry after in gel tryptic digestion of DivIBa and DivIBb.

The optimal 1/100 (w/w) trypsin to protein ratio was first determined based on the appearance of stable fragments of H-DivIB alone. Time course of digestion monitored by Coomassie-stained SDS-PAGE was then performed with the single H-DivIB membrane protein, which revealed an intermediate (DivIBa) and a major (DivIBb) stable fragments predominant after 15 min and 120 min incubation, respectively (Figure 2A). DivIBa and DivIBb fragments migrate on SDS-PAGE as proteins of 45 kDa and 33 kDa in size, respectively, whereas full-length protein has an apparent molecular weight of 56 kDa. N-terminal sequencing performed on both fragments yielded the sequences MGEESE for DivIBa, thus starting at residue M52, and EKPAK for DivIBb, thus starting at residue E116. The two fragments were further characterized by peptide mass fingerprinting. Mass spectrometry after in-gel tryptic digestion identified 6 peptides spanning a region that extends from I123 to K353, for both fragments (Figure 3). Thus, DivIBa and DivIBb fragments span at least residues M52 to K353 and E116 to K353 of pneumococcal DivIB protein, respectively. The β-domain of DivIB was shown previously to resist trypsin digestion up to residue K361 [47]. As peptide mass fingerprinting may not uncover the whole sequence of the protein analysed, it is reasonable to assume that DivIBa covers residues M52-K361, and DivIBb spans residues E116-K361.

Whereas DivIBa retains about half of its cytoplasmic region, DivIBb is truncated of most of its cytoplasmic extremity. Both fragments retain the TM segment, and the α- and β-extracellular domains. These results contrast with the previous structural investigations performed on the recombinant extracellular part DivIB*, which found a trypsin-susceptible unfolded α-domain [47]. The resistance to trypsin digestion of the α-domain in the context of the detergent-solubilized full-length DivIB emphasizes the interest of studying full-length membrane proteins to identify biochemical activities and protein interactions, even in the case of bitopic proteins.

No change in the H-DivIB digestion profile was observed in the presence of DivIC and FtsL (Figure 2B), indicating that the DivIC/FtsL interaction with H-DivIB does not change the accessibility of the tryptic cleavage site of the M1-E116 N-terminal and A362-396 C-terminal parts of the protein. Only the β-domain of DivIB was previously found to interact with DivIC/FtsL in absence of TM segments [47], and to be important for the recruitment of the *E. coli* FtsL/FtsB heterodimer at the division site [52].

The trypsin-digestion profile of H-DivIB was modified in the presence of PBP2x-S (Figure 2C and 2E). Only a small decrease of the intensity of the band corresponding to full length H-DivIB was observed after 120 min of incubation. This result indicates that the whole protein was more resistant to digestion, indicating that the N-terminal cytoplasmic and C-terminal γ-domains of H-DivIB are protected by the interaction with PBP2x-S. The protection of the γ-tail is consistent with the identification of residues close to the C-terminus of DivIB involved in the interaction with the division TP PBP2B in *B. subtilis* [49]. No digestion of H-PBP2x was observed after 120 min of incubation (Figure 2D).

The resistance to trypsin digestion of H-FtsW is also increased when it interacts with DivIB, DivIC, FtsL and PBP2x-S (Figure 2F and 2G), as no degradation product of H-FtsW was observed after 120 min of digestion. Considering the anomalous migration of H-FtsW in SDS-PAGE and the apparent molecular weight of the trypsin-resistant fragment of H-FtsW [26], it is possible that the exposed tryptic cleavage site is located in the large extra-cytoplasmic loop between TM7 and TM8. Given the minimal co-purification of FtsW-S with H-DivIB/DivIC/FtsL in absence of PBP2x-S, and the robust co-purification of H-FtsW with PBP2x-S [32,56], it can be proposed that the large extracellular loop between TM7 and TM8 of H-FtsW is directly involved in the interaction with PBP2x-S. This interpretation would be consistent with the implication of the extra-cellular loop of FtsW of *Mycobacterium tuberculosis* in the interaction with the septal class B PBP [57].

No inhibitory effect of PBP2x-S on trypsin proteolysis was detected with both H-PatAB membrane heterodimer and GST-DivIB* soluble protein (Figure 2H).

Specific PBP2x activity of purified complexes

**Figure 4:**
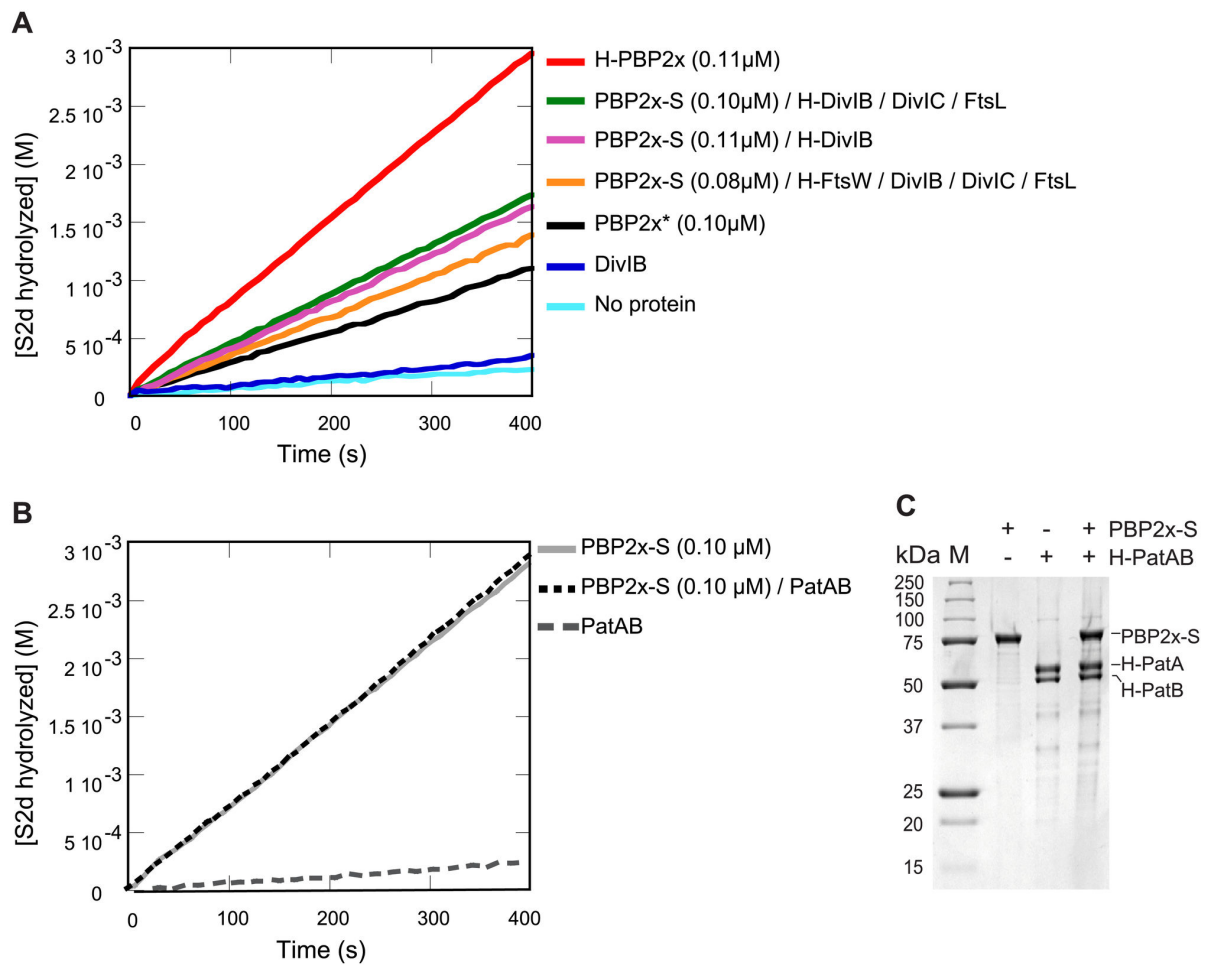
Time course of S2d hydrolysis by purified protein and complexes. **A**. Quantified amount PBP2x active site in the various samples (see Figure S7) is indicated between brackets, **B**. Control experiment of specific PBP2x-S activity in presence or in absence of membrane heterodimer H-PatAB, C. Coomassie-stained SDS-PAGE of PBP2x-S and H-PatAB used in B.

To examine the effect of protein interactions on the accessibility and functionality of the PBP2x active site, we measured its enzymatic activity on the hydrolysis of S2d within the different purified complexes (Figure 4). S2d is a thioesther substrate analogue of the D-Ala-D-Ala-OH terminus of the stem-peptides that are normally cross-linked by the PBPs. The first step of the hydrolysis of S2d mimics the first step of the transpeptidation [44,58].

In order to make meaningful comparisons of the activities, we used the same quantity of H-PBP2x or PBP2X-S in all reactions and determined kinetics parameters (Table 2 and Figure 4). The amount of PBP2x in the various samples was determined by densitometry of gels after SDS-PAGE. The quantification was performed both on Bocillin-stained and Coomassie-stained PBP2x, using the soluble form of PBP2x (PBP2x*) as a standard (Figure S7) [23]. Bocillin is a fluorescent derivative of penicillin. Comparison of the Bocillin- and Coomasssie-stained bands indicated comparable amounts of functional PBP2x active sites in the various preparations. Binding of Bocillin to PBP2x engaged in complexes is a first indication that the TP active site is accessible and in a functional conformation.

**Table 2:** Comparison of kinetic parameter of membrane PBP2x in interaction with membrane proteins involved in septal PG synthesis.

**Protein**	***K*_cat_/*K*_m_ (M^-1^ S^-1^**)**^a^**
PBP2x*	15 000 (± 3 000)
H-PBP2x or PBP2x-S	35 000 (± 3 000)
PBP2x-S/H-DivIB	25 000 (± 2 000)
PBP2x-S/H-DivIB/DivIC/FtsL	22 000 (± 2 000)
PBP2x-S/DivIB/DivIC/FtsL/H-FtsW	25 000 (± 2 000)

^a^ the standard deviation was determined from 4 to 16 independent measurements, * extracellular domain

In our experimental conditions, full-length H-PBP2x or PBP2x-S was two-fold more active than the soluble form PBP2x* (Table 2 and Figure 4A and B). No hydrolysis of S2d was observed in the DDM-solubilized H-DivIB (Figure 4A) or H-PatAB (Figure 4B) preparations. In all complexes, the specific activity of PBP2x-S seemed slightly decreased (with H-DivIB, H-DivIB/DivIC/FtsL, or DivIB/DivIC/FtsL/H-FtsW) compared to the protein alone. No specific activity change of PBP2x-S has been observed in the presence of purified membrane heterodimer H-PatAB (Figure 4B and 4C). The specific small reduction of activity observed with all complexes could be due to a reduction of accessibility of the active site by steric hindrance, or to a down regulation of PBP2x catalytic activity in the presence of partners. We did not consider the possibility of non-specific inhibition, as we did not observed activity variation of PBP2x-S in presence of non-division pneumococcal membrane proteins H-PatAB. Note that S2d is not the physiologically polymerized glycan chain substrate. The effect of the partners of PBP2x-S should ideally be examined on the TP cross-linking of PG and in presence of class A PBP as the cross-linking of the peptide stem should be coordinated with the polymerization of the glycan chain (zapun, unpublished data).

With this work, we presented a strategy that enabled the isolation of membrane protein complexes specifically involved in the cell division of *S. pneumoniae*. This approach should help to identify and purify other complexes comprising Class A PBPs and other conserved division or morphogenetic proteins, which are crucial for understanding the divisome. Our study constitutes the necessary first step toward the *in vitro* reconstitution of septal PG synthesis. The successful purification of several membrane complexes involved in this process will help to understand the role of the central DivIB/DivIC/FtsL proteins, as soon as optimal conditions of septal PG assembly will be identified for *S. pneumoniae*.

## Supporting Information

Figure S1 **Detergent screening for the solubilization and purification of the H-DivIB/DivIC/FtsL membrane protein complex.** Purified membranes from *E. coli* cells expressing the three proteins were solubilized with 22 mM DM, 5 mM DDM, 38 mM β-D-octyl glucoside (B-OG), 21.5 mM 3-laurylamido-N,N’-dimethylpropyl amine oxide (LAPAO), 42 mM NM, 4.5 mM lauryl maltose neopentyl glycol (MNG3), 21 mM lauryldimethylamine-N-oxide (LDAO), 28 mM 3-[(3-cholamidopropyl) dimethylammonio]-1-propanesulfonate (CHAPS), 22.5 mM 5-cyclohexyl-1-pentyl-β-D-maltoside (Cymal 5), 21.5 mM n-dodecylphosphocholine (Fos-choline 12, FC-12), 20 mM Triton X-100. After Ni-NTA affinity chromatography, the purification of H-DivIB and the co-purification of DivIC and FtsL was analyzed by Coomassie-stained SDS-PAGE.
(TIF)

Figure S2 **Comparative purification of H-DivIB/DivIC/FtsL/PBP2x-S and H-DivIB/PBP2x-S membrane protein complexes.** Membrane preparations of *E. coli* strains overexpressing pneumococcal membrane proteins were subjected to detergent solubilization, Ni-NTA and Strep-Tactin affinity chromatography. L, W, E and Econc stand for load, wash, elution and concentrated elution fractions, respectively. Samples were analysed by Coomassie-stained SDS-PAGE.
(TIF)

Figure S3 Interaction of the extracellular domains PBP2x* and DivIB* with full length H-DivIB and PBP2x-S membrane proteins, respectively. Cytoplasmic fraction of *E. coli* strains overexpressing GST-fusion proteins mixed to solubilized membrane preparations, were subjected to Ni-NTA, or Strep-Tactin followed by glutathion affinity chromatography. (A) GST-PBP2x*, H-DivIB; (B) GST-PBP2x*; (C) GST-DivIB*, PBP2x-S. Samples were analysed by Coomassie-stained SDS PAGES L, W and E stand for load, wash and elution fractions, respectively. Ni-NTA, Strep and GSH stand for Ni-NTA, Strep-Tactin and glutathion Sepharose affinity chromatography, respectively.
(TIF)

Figure S4 **Identification of membrane proteins involved in purified complexes by Western blot.** The identity of each protein in purified membrane protein complexes was checked by Western blot using specific antibodies against DivIB, DivIC, FtsL, PBP2x proteins, and His- or Strep-tag. CB stands for Coomassie blue staining, WB stands for Western blot. E Ni-NTA and E Strep stand for elution from Ni-NTA or Strep-Tactin chromatography.
(TIF)

Figure S5 **Controls of the specificity of the Ni-NTA chromatography and affinity purification of the individual membrane proteins H-DivIB, H-PBP2x or H-FtsW.** Membrane preparation of *E. coli* strains overexpressing proteins without His-tag DivIB, DivIC, FtsL, PBP2x-S and FtsW (**A**), or His-tagged membrane proteins H-DivIB (**B**), H-PBP2x (**C**), or H-FtsW (**D**), were subjected to detergent solubilisation and Ni-NTA chromatography. Samples were analysed by Coomassie-stained SDS PAGES L, W and E stand for load, wash and elution fractions, respectively. Ni-NTA and Strep stand for Ni-NTA or Strep-Tactin chromatography.
(TIF)

Figure S6 **Gel filtration chromatograms of the membrane protein H-DivIB or purified septal membrane protein complexes.** Proteins were purified by Ni-NTA and Strep-Tactin affinity chromatography and concentrated before analysis by size exclusion chromatography on Superdex 200 (**A**) or Superose 6 (**B**) matrixes.
(TIF)

Figure S7 **Determination of full length PBP2x concentration, alone and in complexes, by Bocillin-FL- and Coomassie-stained SDS-PAGE.** Standards were varying concentrations of the soluble form PBP2x*. Concentrations of PBP2x were determined independently from two volumes of protein.
(TIF)

## References

[1] Martos A, Jiménez M, Rivas G, Schwille P (2012) Towards a bottom-up reconstitution of bacterial cell division. Trends Cell Biol 22: 634-643. doi:10.1016/j.tcb.2012.09.003. PubMed: 23067680. 23067680

[2] Lebar MD, Lupoli TJ, Tsukamoto H, May JM, Walker S et al. (2013) Forming Cross-Linked Peptidoglycan from Synthetic Gram-Negative Lipid II. J Am Chem Soc 135: 4632-4635. doi:10.1021/ja312510m. PubMed: 23480167. 23480167 PMC3658469

[3] Typas A, Banzhaf M, Gross CA, Vollmer W (2012) From the regulation of peptidoglycan synthesis to bacterial growth and morphology. Nat Rev Microbiol 10: 123-136. PubMed: 22203377. 10.1038/nrmicro2677 PMC5433867 22203377

[4] Ghuysen JM (1968) Use of bacteriolytic enzymes in determination of wall structure and their role in cell metabolism. Bacteriol Rev 32: 425-464. PubMed: 4884715. 4884715 PMC413160

[5] Schleifer KH, Kandler O (1972) Peptidoglycan types of bacterial cell walls and their taxonomic implications. Bacteriol Rev 36: 407-477. PubMed: 4568761. 4568761 10.1128/br.36.4.407-477.1972 PMC408328

[6] Vollmer W, Blanot D, de Pedro MA (2008) Peptidoglycan structure and architecture. FEMS Microbiol Rev 32: 149-167. doi:10.1111/j.1574-6976.2007.00094.x. PubMed: 18194336. 18194336

[7] Höltje JV (1998) Growth of the stress-bearing and shape-maintaining murein sacculus of Escherichia coli. Microbiol Mol Biol Rev 62: 181-203. PubMed: 9529891. 9529891 10.1128/mmbr.62.1.181-203.1998 PMC98910

[8] Cabeen MT, Jacobs-Wagner C (2007) Skin and bones: the bacterial cytoskeleton, cell wall, and cell morphogenesis. J Cell Biol 179: 381-387. doi:10.1083/jcb.200708001. PubMed: 17967949. 17967949 PMC2064785

[9] Bouhss A, Trunkfield AE, Bugg TD, Mengin-Lecreulx D (2008) The biosynthesis of peptidoglycan lipid-linked intermediates. FEMS Microbiol Rev 32: 208-233. doi:10.1111/j.1574-6976.2007.00089.x. PubMed: 18081839. 18081839

[10] den Blaauwen T, de Pedro MA, Nguyen-Distèche M, Ayala JA (2008) Morphogenesis of rod-shaped sacculi. FEMS Microbiol Rev 32: 321-344. doi:10.1111/j.1574-6976.2007.00090.x. PubMed: 18291013. 18291013

[11] Banzhaf M, van den Berg van Saparoea B, Terrak M, Fraipont C, Egan A et al. (2012) Cooperativity of peptidoglycan synthases active in bacterial cell elongation. Mol Microbiol 85: 179-194. doi:10.1111/j.1365-2958.2012.08103.x. PubMed: 22606933. 22606933

[12] Bertsche U, Breukink E, Kast T, Vollmer W (2005) In vitro murein peptidoglycan synthesis by dimers of the bifunctional transglycosylase-transpeptidase PBP1B from Escherichia coli. J Biol Chem 280: 38096-38101. doi:10.1074/jbc.M508646200. PubMed: 16154998. 16154998

[13] Born P, Breukink E, Vollmer W (2006) In vitro synthesis of cross-linked murein and its attachment to sacculi by PBP1A from Escherichia coli. J Biol Chem 281: 26985-26993. doi:10.1074/jbc.M604083200. PubMed: 16840781. 16840781

[14] Helassa N, Vollmer W, Breukink E, Vernet T, Zapun A (2012) The membrane anchor of penicillin-binding protein PBP2a from Streptococcus pneumoniae influences peptidoglycan chain length. FEBS J 279: 2071-2081. doi:10.1111/j.1742-4658.2012.08592.x. PubMed: 22487093. 22487093

[15] Zapun A, Contreras-Martel C, Vernet T (2008) Penicillin-binding proteins and beta-lactam resistance. FEMS Microbiol Rev 32: 361-385. doi:10.1111/j.1574-6976.2007.00095.x. PubMed: 18248419. 18248419

[16] Pérez-Núñez D, Briandet R, David B, Gautier C, Renault P et al. (2011) A new morphogenesis pathway in bacteria: unbalanced activity of cell wall synthesis machineries leads to coccus-to-rod transition and filamentation in ovococci. Mol Microbiol 79: 759-771. doi:10.1111/j.1365-2958.2010.07483.x. PubMed: 21255117. 21255117

[17] van Opijnen T, Bodi KL, Camilli A (2009) Tn-seq: high-throughput parallel sequencing for fitness and genetic interaction studies in microorganisms. Nat Methods 6: 767-772. doi:10.1038/nmeth.1377. PubMed: 19767758. 19767758 PMC2957483

[18] Zapun A, Vernet T, Pinho MG (2008) The different shapes of cocci. FEMS Microbiol Rev 32: 345-360. doi:10.1111/j.1574-6976.2007.00098.x. PubMed: 18266741. 18266741

[19] Sham LT, Tsui HC, Land AD, Barendt SM, Winkler ME (2012) Recent advances in pneumococcal peptidoglycan biosynthesis suggest new vaccine and antimicrobial targets. Curr Opin Microbiol 15: 194-203. doi:10.1016/j.mib.2011.12.013. PubMed: 22280885. 22280885 PMC3322672

[20] Land AD, Winkler ME (2011) The requirement for pneumococcal MreC and MreD is relieved by inactivation of the gene encoding PBP1a. J Bacteriol 193: 4166-4179. doi:10.1128/JB.05245-11. PubMed: 21685290. 21685290 PMC3147673

[21] Claessen D, Emmins R, Hamoen LW, Daniel RA, Errington J et al. (2008) Control of the cell elongation-division cycle by shuttling of PBP1 protein in Bacillus subtilis. Mol Microbiol 68: 1029-1046. doi:10.1111/j.1365-2958.2008.06210.x. PubMed: 18363795. 18363795

[22] Massidda O, Nováková L, Vollmer W (2013) From models to pathogens: how much have we learned about Streptococcus pneumoniae cell division? Environ Microbiol: ([MedlinePgn:]) doi:10.1111/1462-2920.12189. PubMed: 23848140. 23848140

[23] Pares S, Mouz N, Pétillot Y, Hakenbeck R, Dideberg O (1996) X-ray structure of Streptococcus pneumoniae PBP2x, a primary penicillin target enzyme. Nat Struct Biol 3: 284-289. doi:10.1038/nsb0396-284. PubMed: 8605631. 8605631

[24] Margolin W (2000) Themes and variations in prokaryotic cell division. FEMS Microbiol Rev 24: 531-548. doi:10.1111/j.1574-6976.2000.tb00554.x. PubMed: 10978550. 10978550

[25] Buddelmeijer N, Beckwith J (2004) A complex of the Escherichia coli cell division proteins FtsL, FtsB and FtsQ forms independently of its localization to the septal region. Mol Microbiol 52: 1315-1327. doi:10.1111/j.1365-2958.2004.04044.x. PubMed: 15165235. 15165235

[26] Noirclerc-Savoye M, Le Gouëllec A, Morlot C, Dideberg O, Vernet T et al. (2005) In vitro reconstitution of a trimeric complex of DivIB, DivIC and FtsL, and their transient co-localization at the division site in Streptococcus pneumoniae. Mol Microbiol 55: 413-424. PubMed: 15659160. 15659160 10.1111/j.1365-2958.2004.04408.x

[27] Gérard P, Vernet T, Zapun A (2002) Membrane topology of the Streptococcus pneumoniae FtsW division protein. J Bacteriol 184: 1925-1931. doi:10.1128/JB.184.7.1925-1931.2002. PubMed: 11889099. 11889099 PMC134934

[28] Mohammadi T, van Dam V, Sijbrandi R, Vernet T, Zapun A et al. (2011) Identification of FtsW as a transporter of lipid-linked cell wall precursors across the membrane. EMBO J 30: 1425-1432. doi:10.1038/emboj.2011.61. PubMed: 21386816. 21386816 PMC3102273

[29] Robichon C, King GF, Goehring NW, Beckwith J (2008) Artificial septal targeting of Bacillus subtilis cell division proteins in Escherichia coli: an interspecies approach to the study of protein-protein interactions in multiprotein complexes. J Bacteriol 190: 6048-6059. doi:10.1128/JB.00462-08. PubMed: 18621900. 18621900 PMC2546800

[30] Errington J, Daniel RA, Scheffers DJ (2003) Cytokinesis in bacteria. Microbiol Mol Biol Rev 67: 52-65. doi:10.1128/MMBR.67.1.52-65.2003. PubMed: 12626683. 12626683 PMC150516

[31] Gamba P, Veening JW, Saunders NJ, Hamoen LW, Daniel RA (2009) Two-step assembly dynamics of the Bacillus subtilis divisome. J Bacteriol 191: 4186-4194. doi:10.1128/JB.01758-08. PubMed: 19429628. 19429628 PMC2698510

[32] Fraipont C, Alexeeva S, Wolf B, van der Ploeg R, Schloesser M et al. (2011) The integral membrane FtsW protein and peptidoglycan synthase PBP3 form a subcomplex in Escherichia coli. Microbiology 157: 251-259. doi:10.1099/mic.0.040071-0. PubMed: 20847002. 20847002

[33] D’Ulisse V, Fagioli M, Ghelardini P, Paolozzi L (2007) Three functional subdomains of the Escherichia coli FtsQ protein are involved in its interaction with the other division proteins. Microbiology 153: 124-138. doi:10.1099/mic.0.2006/000265-0. PubMed: 17185541. 17185541

[34] Arends SJ, Weiss DS (2004) Inhibiting cell division in Escherichia coli has little if any effect on gene expression. J Bacteriol 186: 880-884. doi:10.1128/JB.186.3.880-884.2004. PubMed: 14729718. 14729718 PMC321490

[35] Wadsworth KD, Rowland SL, Harry EJ, King GF (2008) The divisomal protein DivIB contains multiple epitopes that mediate its recruitment to incipient division sites. Mol Microbiol 67: 1143-1155. doi:10.1111/j.1365-2958.2008.06114.x. PubMed: 18208530. 18208530

[36] Weiss DS, Chen JC, Ghigo JM, Boyd D, Beckwith J (1999) Localization of FtsI (PBP3) to the septal ring requires its membrane anchor, the Z ring, FtsA, FtsQ, and FtsL. J Bacteriol 181: 508-520. 9882665 10.1128/jb.181.2.508-520.1999 PMC93405

[37] Piette A, Fraipont C, Den Blaauwen T, Aarsman ME, Pastoret S et al. (2004) Structural determinants required to target penicillin-binding protein 3 to the septum of Escherichia coli. J Bacteriol 186: 6110-6117. doi:10.1128/JB.186.18.6110-6117.2004. PubMed: 15342580. 15342580 PMC515155

[38] Wissel MC, Weiss DS (2004) Genetic analysis of the cell division protein FtsI (PBP3): amino acid substitutions that impair septal localization of FtsI and recruitment of FtsN. J Bacteriol 186: 490-502. doi:10.1128/JB.186.2.490-502.2004. PubMed: 14702319. 14702319 PMC305773

[39] Wissel MC, Wendt JL, Mitchell CJ, Weiss DS (2005) The transmembrane helix of the Escherichia coli division protein FtsI localizes to the septal ring. J Bacteriol 187: 320-328. doi:10.1128/JB.187.1.320-328.2005. PubMed: 15601716. 15601716 PMC538840

[40] Ghigo JM, Beckwith J (2000) Cell division in Escherichia coli: role of FtsL domains in septal localization, function, and oligomerization. J Bacteriol 182: 116-129. doi:10.1128/JB.182.1.116-129.2000. PubMed: 10613870. 10613870 PMC94247

[41] Noirclerc-Savoye M, Gallet B, Bernaudat F, Vernet T (2010) Large scale purification of linear plasmid DNA for efficient high throughput cloning. Biotechnol J 5: 978-985. doi:10.1002/biot.201000132. PubMed: 20845387. 20845387

[42] Boncoeur E, Durmort C, Bernay B, Ebel C, Di Guilmi AM et al. (2012) PatA and PatB form a functional heterodimeric ABC multidrug efflux transporter responsible for the resistance of Streptococcus pneumoniae to fluoroquinolones. Biochemistry 51: 7755-7765. doi:10.1021/bi300762p. PubMed: 22950454. 22950454

[43] Rosenfeld J, Capdevielle J, Guillemot JC, Ferrara P (1992) In-gel digestion of proteins for internal sequence analysis after one- or two-dimensional gel electrophoresis. Anal Biochem 203: 173-179. doi:10.1016/0003-2697(92)90061-B. PubMed: 1524213. 1524213

[44] Zhao G, Yeh WK, Carnahan RH, Flokowitsch J, Meier TI et al. (1997) Biochemical characterization of penicillin-resistant and -sensitive penicillin-binding protein 2x transpeptidase activities of Streptococcus pneumoniae and mechanistic implications in bacterial resistance to beta-lactam antibiotics. J Bacteriol 179: 4901-4908. PubMed: 9244281. 9244281 10.1128/jb.179.15.4901-4908.1997 PMC179340

[45] di Guilmi AM, Mouz N, Martin L, Hoskins J, Jaskunas SR et al. (1999) Glycosyltransferase domain of penicillin-binding protein 2a from Streptococcus pneumoniae is membrane associated. J Bacteriol 181: 2773-2781. PubMed: 10217767. 10217767 10.1128/jb.181.9.2773-2781.1999 PMC93718

[46] Berridge G, Chalk R, D’Avanzo N, Dong L, Doyle D et al. (2011) High-performance liquid chromatography separation and intact mass analysis of detergent-solubilized integral membrane proteins. Anal Biochem 410: 272-280. doi:10.1016/j.ab.2010.11.008. PubMed: 21093405. 21093405 PMC3041925

[47] Masson S, Kern T, Le Gouëllec A, Giustini C, Simorre JP et al. (2009) Central domain of DivIB caps the C-terminal regions of the FtsL/DivIC coiled-coil rod. J Biol Chem 284: 27687-27700. doi:10.1074/jbc.M109.019471. PubMed: 19635793. 19635793 PMC2785697

[48] Berg KH, Stamsas GA, Straume D, Havarstein LS (2013) The effect of low Pbp2b levels on cell morphology and peptidoglycan composition in Streptococcus pneumoniae R6. J Bacteriol. doi:10.1128/JB.00184-13. PMC3807472 23873916

[49] Rowland SL, Wadsworth KD, Robson SA, Robichon C, Beckwith J et al. (2010) Evidence from artificial septal targeting and site-directed mutagenesis that residues in the extracytoplasmic β domain of DivIB mediate its interaction with the divisomal transpeptidase PBP 2B. J Bacteriol 192: 6116-6125. doi:10.1128/JB.00783-10. PubMed: 20870765. 20870765 PMC2981201

[50] Mercer KL, Weiss DS (2002) The Escherichia coli cell division protein FtsW is required to recruit its cognate transpeptidase, FtsI (PBP3), to the division site. J Bacteriol 184: 904-912. doi:10.1128/jb.184.4.904-912.2002. PubMed: 11807049. 11807049 PMC134820

[51] Massidda O, Anderluzzi D, Friedli L, Feger G (1998) Unconventional organization of the division and cell wall gene cluster of Streptococcus pneumoniae. Microbiology 144 ( Pt 11): 3069-3078. doi:10.1099/00221287-144-11-3069. PubMed: 9846742. 9846742

[52] van den Ent F, Vinkenvleugel TM, Ind A, West P, Veprintsev D et al. (2008) Structural and mutational analysis of the cell division protein FtsQ. Mol Microbiol 68: 110-123. doi:10.1111/j.1365-2958.2008.06141.x. PubMed: 18312270. 18312270

[53] Lapointe LM, Taylor KC, Subramaniam S, Khadria A, Rayment I et al. (2013) Structural organization of FtsB, a transmembrane protein of the bacterial divisome. Biochemistry, 52: 2574–85. doi:10.1021/bi400222r. PubMed: 23520975. 23520975 PMC3702382

[54] Korepanova A, Matayoshi ED (2012) HPLC-SEC characterization of membrane protein-detergent complexes. Curr Protoc Protein Sci Chapter 29: Unit 29.5.1: 21-12 PubMed: 22470129. 10.1002/0471140864.ps2905s68 22470129

[55] Villanelo F, Ordenes A, Brunet J, Lagos R, Monasterio O (2011) A model for the Escherichia coli FtsB/FtsL/FtsQ cell division complex. BMC Struct Biol 11: 28. doi:10.1186/1472-6807-11-28. PubMed: 21672257. 21672257 PMC3152878

[56] Zapun A, Noirclerc-Savoye M, Helassa N, Vernet T (2012) Peptidoglycan assembly machines: the biochemical evidence. Microb Drug Resist 18: 256-260. doi:10.1089/mdr.2011.0236. PubMed: 22432702. 22432702

[57] Datta P, Dasgupta A, Singh AK, Mukherjee P, Kundu M et al. (2006) Interaction between FtsW and penicillin-binding protein 3 (PBP3) directs PBP3 to mid-cell, controls cell septation and mediates the formation of a trimeric complex involving FtsZ, FtsW and PBP3 in mycobacteria. Mol Microbiol 62: 1655-1673. doi:10.1111/j.1365-2958.2006.05491.x. PubMed: 17427288. 17427288

[58] Adam M, Damblon C, Jamin M, Zorzi W, Dusart V et al. (1991) Acyltransferase activities of the high-molecular-mass essential penicillin-binding proteins. Biochem J 279(2): 601-604. PubMed: 1953655. 1953655 10.1042/bj2790601 PMC1151646

