# Supplementary figures and images for: Reconstitution of Membrane Protein Complexes Involved in Pneumococcal Septal Cell Wall Assembly
Source: PLoS One. 2013 Sep 23;8(9):e75522. doi: 10.1371/journal.pone.0075522 (PMC3798694; doi:10.1371/journal.pone.0075522)

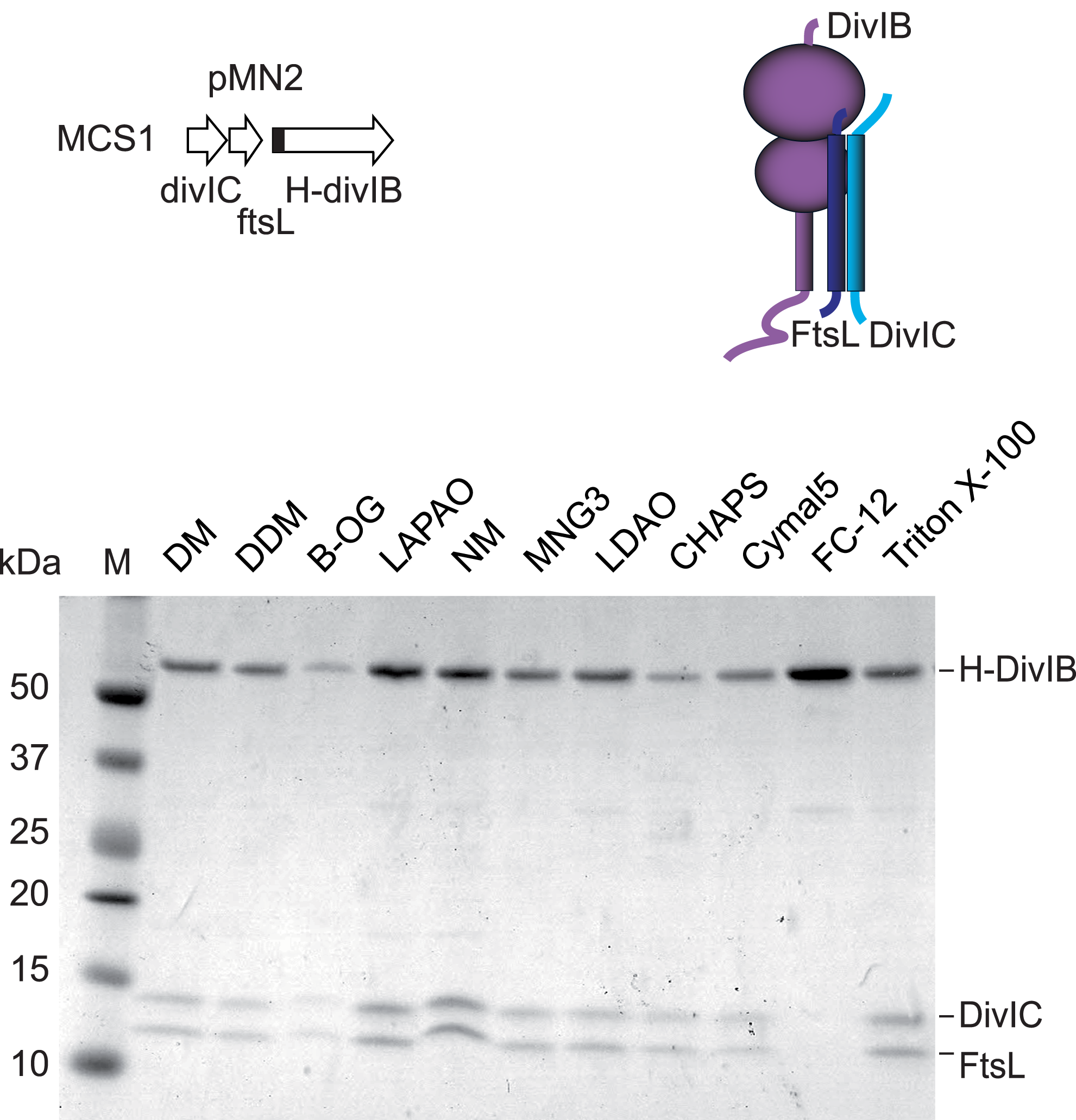

Supplement: Figure S1 — Detergent screening for the solubilization and purification of the H-DivIB/DivIC/FtsL membrane protein complex. Purified membranes from E. coli cells expressing the three proteins were solubilized with 22 mM DM, 5 mM DDM, 38 mM β-D-octyl glucoside (B-OG), 21.5 mM 3-laurylamido-N,N’-dimethylpropyl amine oxide (LAPAO), 42 mM NM, 4.5 mM lauryl maltose neopentyl glycol (MNG3), 21 mM lauryldimethylamine-N-oxide (LDAO), 28 mM 3-[(3-cholamidopropyl) dimethylammonio]-1-propanesulfonate (CHAPS), 22.5 mM 5-cyclohexyl-1-pentyl-β-D-maltoside (Cymal 5), 21.5 mM n-dodecylphosphocholine (Fos-choline 12, FC-12), 20 mM Triton X-100. After Ni-NTA affinity chromatography, the purification of H-DivIB and the co-purification of DivIC and FtsL was analyzed by Coomassie-stained SDS-PAGE. (TIF) [file pone.0075522.s001.tif]

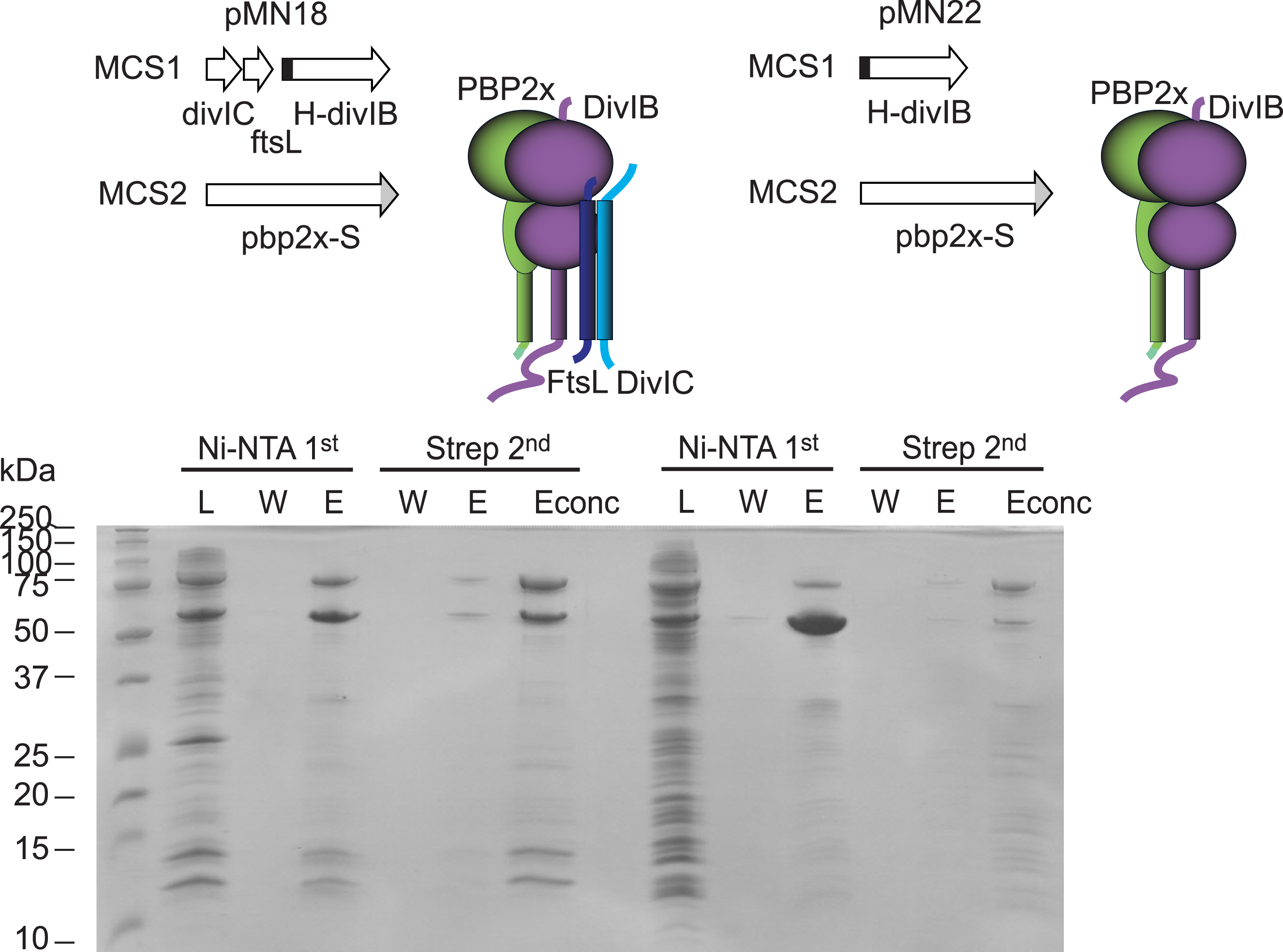

Supplement: Figure S2 — Comparative purification of H-DivIB/DivIC/FtsL/PBP2x-S and H-DivIB/PBP2x-S membrane protein complexes. Membrane preparations of E. coli strains overexpressing pneumococcal membrane proteins were subjected to detergent solubilization, Ni-NTA and Strep-Tactin affinity chromatography. L, W, E and Econc stand for load, wash, elution and concentrated elution fractions, respectively. Samples were analysed by Coomassie-stained SDS-PAGE. (TIF) [file pone.0075522.s002.tif]

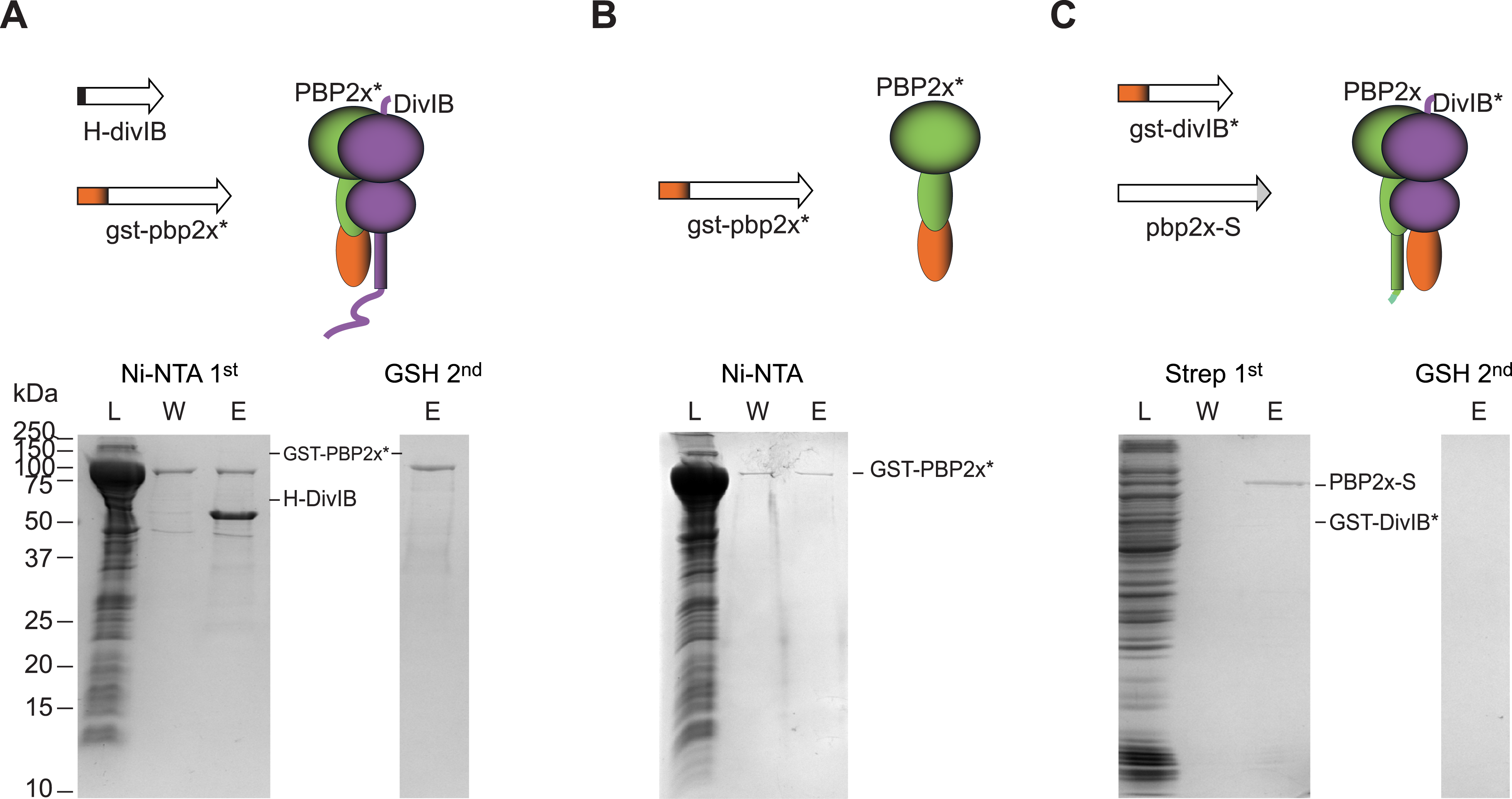

Supplement: Figure S3 — Interaction of the extracellular domains PBP2x* and DivIB* with full length H-DivIB and PBP2x-S membrane proteins, respectively. Cytoplasmic fraction of E. coli strains overexpressing GST-fusion proteins mixed to solubilized membrane preparations, were subjected to Ni-NTA, or Strep-Tactin followed by glutathion affinity chromatography. (A) GST-PBP2x*, H-DivIB; (B) GST-PBP2x*; (C) GST-DivIB*, PBP2x-S. Samples were analysed by Coomassie-stained SDS PAGES L, W and E stand for load, wash and elution fractions, respectively. Ni-NTA, Strep and GSH stand for Ni-NTA, Strep-Tactin and glutathion Sepharose affinity chromatography, respectively. (TIF) [file pone.0075522.s003.tif]

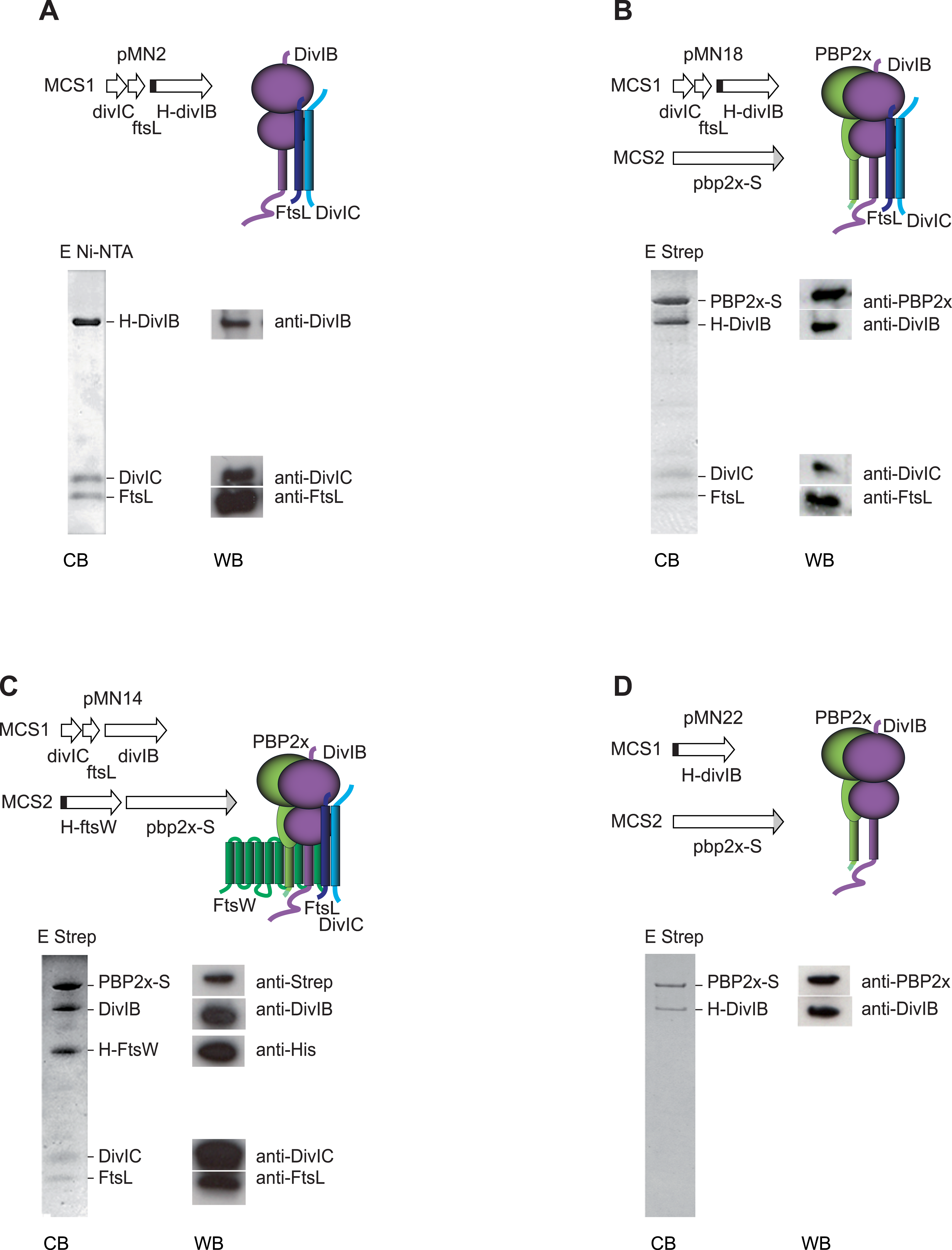

Supplement: Figure S4 — Identification of membrane proteins involved in purified complexes by Western blot. The identity of each protein in purified membrane protein complexes was checked by Western blot using specific antibodies against DivIB, DivIC, FtsL, PBP2x proteins, and His- or Strep-tag. CB stands for Coomassie blue staining, WB stands for Western blot. E Ni-NTA and E Strep stand for elution from Ni-NTA or Strep-Tactin chromatography. (TIF) [file pone.0075522.s004.tif]

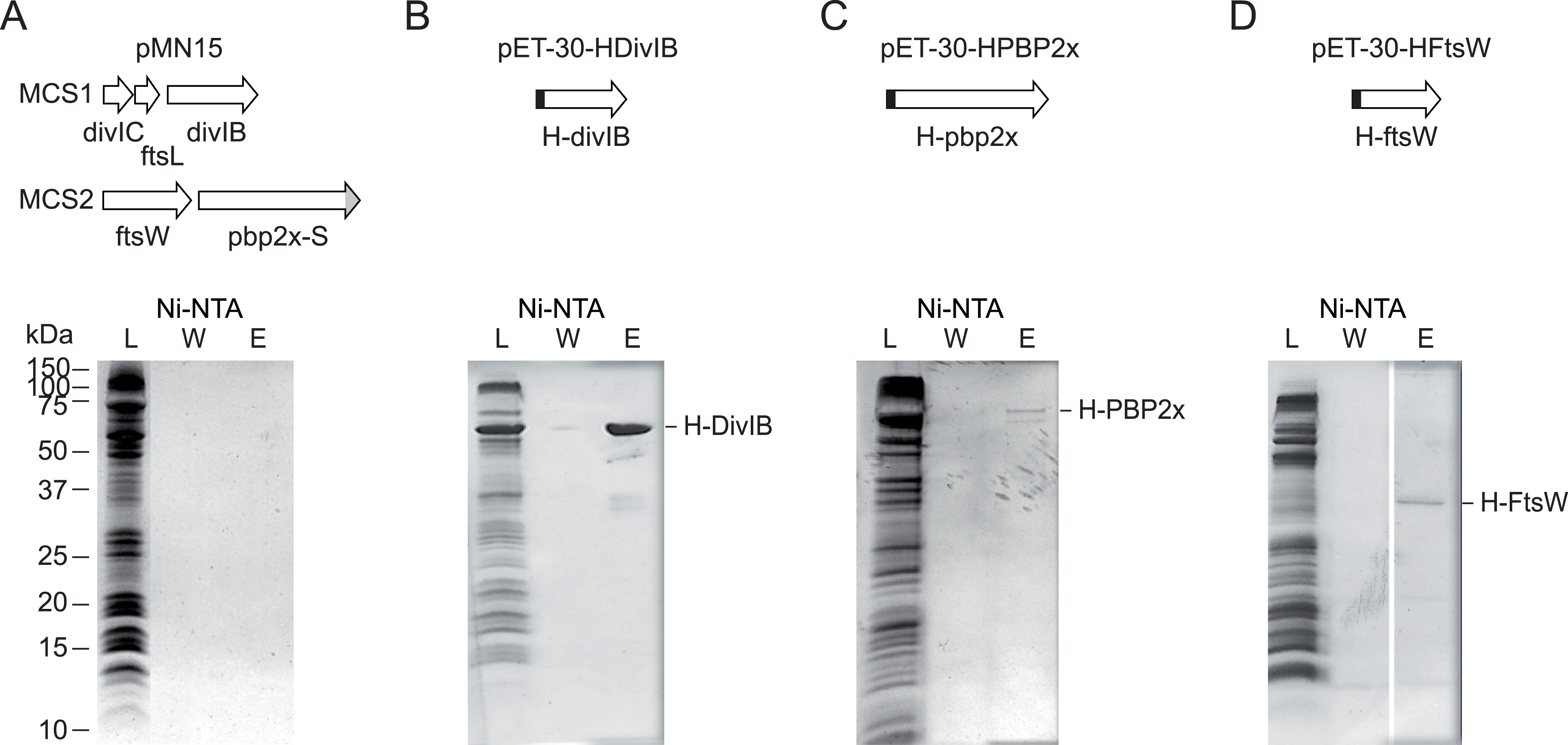

Supplement: Figure S5 — Controls of the specificity of the Ni-NTA chromatography and affinity purification of the individual membrane proteins H-DivIB, H-PBP2x or H-FtsW. Membrane preparation of E. coli strains overexpressing proteins without His-tag DivIB, DivIC, FtsL, PBP2x-S and FtsW (A), or His-tagged membrane proteins H-DivIB (B), H-PBP2x (C), or H-FtsW (D), were subjected to detergent solubilisation and Ni-NTA chromatography. Samples were analysed by Coomassie-stained SDS PAGES L, W and E stand for load, wash and elution fractions, respectively. Ni-NTA and Strep stand for Ni-NTA or Strep-Tactin chromatography. (TIF) [file pone.0075522.s005.tif]

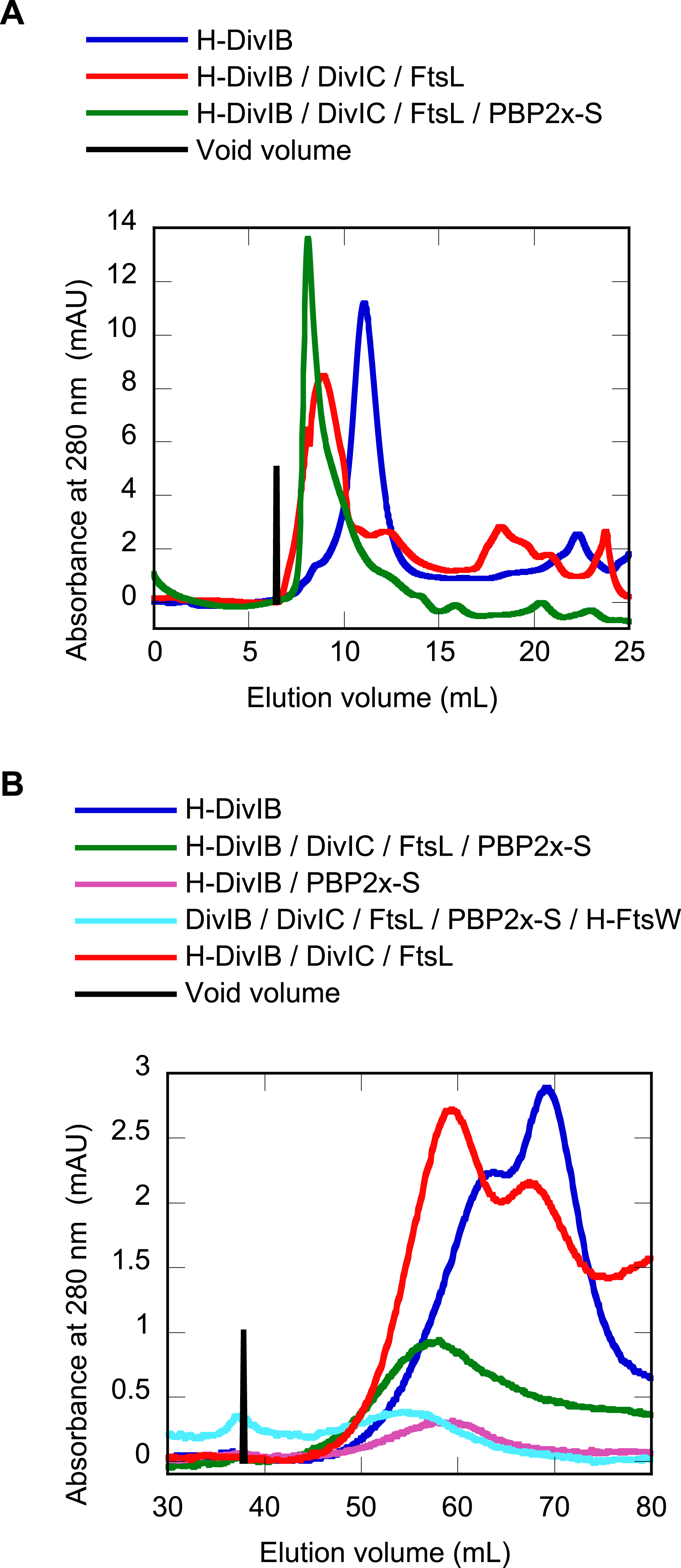

Supplement: Figure S6 — Gel filtration chromatograms of the membrane protein H-DivIB or purified septal membrane protein complexes. Proteins were purified by Ni-NTA and Strep-Tactin affinity chromatography and concentrated before analysis by size exclusion chromatography on Superdex 200 (A) or Superose 6 (B) matrixes. (TIF) [file pone.0075522.s006.tif]

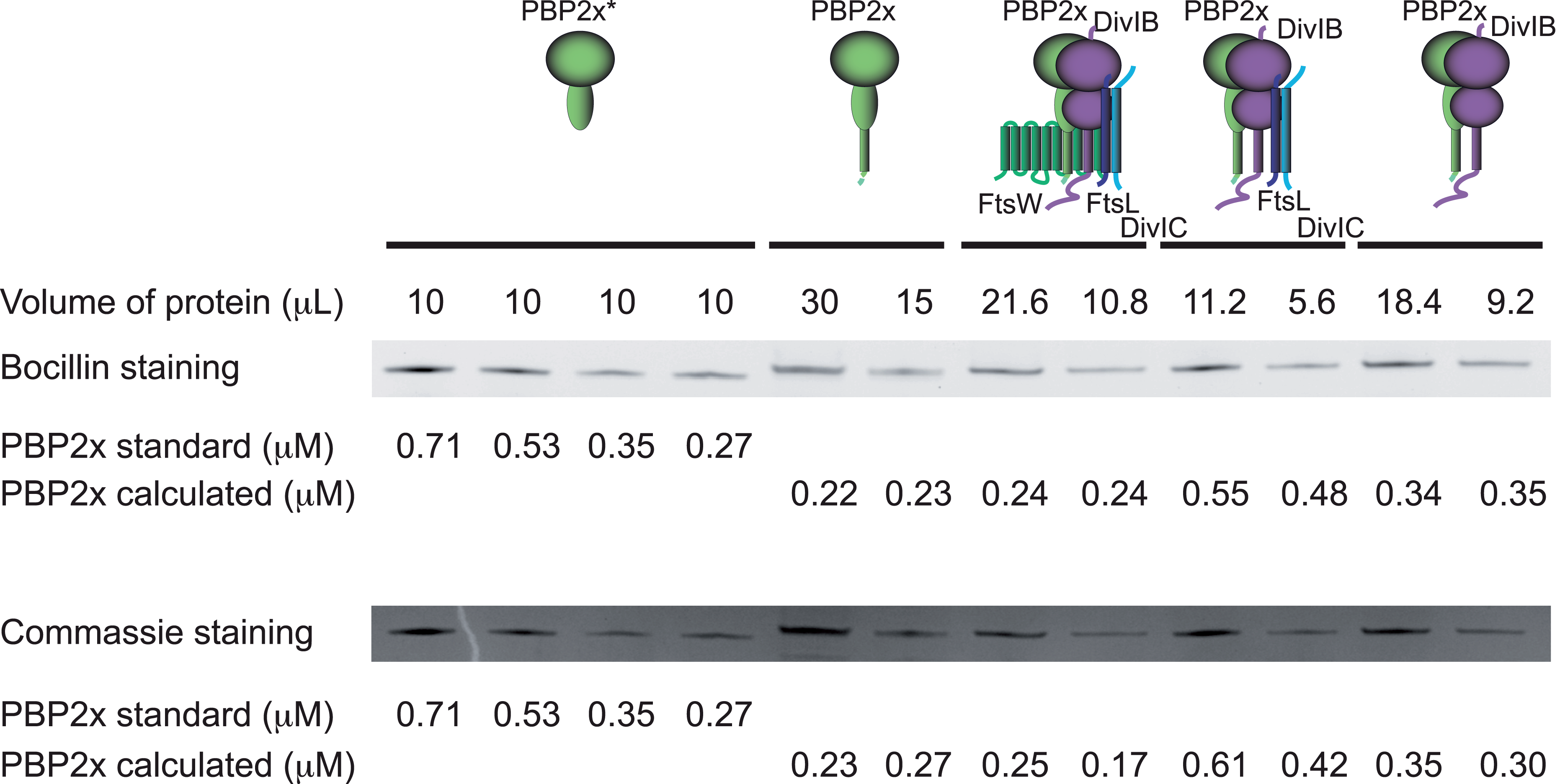

Supplement: Figure S7 — Determination of full length PBP2x concentration, alone and in complexes, by Bocillin-FL- and Coomassie-stained SDS-PAGE. Standards were varying concentrations of the soluble form PBP2x*. Concentrations of PBP2x were determined independently from two volumes of protein. (TIF) [file pone.0075522.s007.tif]
